# A first-generation integrated tammar wallaby map and its use in creating a tammar wallaby first-generation virtual genome map

**DOI:** 10.1186/1471-2164-12-422

**Published:** 2011-08-19

**Authors:** Chenwei Wang, Janine E Deakin, Willem Rens, Kyall R Zenger, Katherine Belov, Jennifer A Marshall Graves, Frank W Nicholas

**Affiliations:** 1 Australian Research Council (ARC) Centre of Excellence for Kangaroo Genomics; 2 Faculty of Veterinary Science, University of Sydney, NSW 2006, Australia; 3 Research School of Biology, The Australian National University, Canberra, ACT 0200, Australia; 4 Department of Veterinary Medicine, University of Cambridge, UK; 5 School of Marine & Tropical Biology, James Cook University, Townsville, QLD 4811, Australia

## Abstract

**Background:**

The limited (2X) coverage of the tammar wallaby (*Macropus eugenii*) genome sequence dataset currently presents a challenge for assembly and anchoring onto chromosomes. To provide a framework for this assembly, it would be a great advantage to have a dense map of the tammar wallaby genome. However, only limited mapping data are available for this non-model species, comprising a physical map and a linkage map.

**Results:**

We combined all available tammar wallaby mapping data to create a tammar wallaby integrated map, using the Location DataBase (LDB) strategy. This first-generation integrated map combines all available information from the second-generation tammar wallaby linkage map with 148 loci, and extensive FISH mapping data for 492 loci, especially for genes likely to be located at the ends of wallaby chromosomes or at evolutionary breakpoints inferred from comparative information. For loci whose positions are only approximately known, their location in the integrated map was refined on the basis of comparative information from opossum (*Monodelphis domestica*) and human. Interpolation of segments from the opossum and human assemblies into the integrated map enabled the subsequent construction of a tammar wallaby first-generation virtual genome map, which comprises 14336 markers, including 13783 genes recruited from opossum and human assemblies. Both maps are freely available at http://compldb.angis.org.au.

**Conclusions:**

The first-generation integrated map and the first-generation virtual genome map provide a backbone for the chromosome assembly of the tammar wallaby genome sequence. For example, 78% of the 10257 gene-scaffolds in the Ensembl annotation of the tammar wallaby genome sequence (including 10522 protein-coding genes) can now be given a chromosome location in the tammar wallaby virtual genome map.

## Background

Stimulated by the human genome project and the need to further understand species genome architecture, more divergent mammals are being sequenced [1-7], providing more complete and detailed comparative genomic information, and contributing to our understanding of mammalian genome evolution.

Marsupials are a particularly interesting lineage of mammals, being only distantly related to eutherian (placental) mammals such as human and mouse. They have many major features common to all mammals, such as bearing fur and suckling young, but they show many unique characteristics in reproduction, lactation, sex determination and immunology [8]. Marsupials last shared a common ancestor with eutherians about 150 million years ago (mya) [9], an evolutionary distance sufficient to exclude the conservation of most non-functional sequence, but not too wide to lose the conservation of most functional sequence [10].

Two marsupial genomes have been sequenced to date, representing lineages of marsupials living in South America and Australia that diverged about 70 mya [11]. The Brazilian opossum (*Monodelphis domestica*) was sequenced with more than 7× coverage in 2007 [5]. The Australian model kangaroo, the tammar wallaby (*Macropus eugenii*), has recently been sequenced at about 2× coverage [12]. The first version (Meug_1.0) of the tammar wallaby assembly, released in December 2008, comprised relatively small contigs (N50 = 2.5 kb) and scaffolds (14.5 kb), consistent with the relatively low coverage. In July 2009, Ensembl released its annotation of these segments [13], but there remains insufficient genome structure information to enable any of these sequence segments to be assembled onto *M. eugenii *chromosomes.

The situation is comparable to that of genomes of other species sequenced at low coverage. Sequence (1.9×) of the domestic cat (*Felis catus*) [14] was assembled first by reconstituting cat contigs on the basis of their alignment with the dog assembly, then aligning these revised contigs with a cat radiation hybrid (RH) map. Although this approach was productive, it largely neglected other cat mapping resources such as the cat linkage map. The sheep genome, too, has been sequenced to approximately 2× coverage (primarily for SNP discovery) [15]. In this species, great use was made of sheep BAC-end sequences (BES), which were assembled in a consensus manner based on their alignments to cattle, human and dog sequence assemblies. A virtual sheep genome was then created by transferring relevant human assembly segments onto the consensus alignment of sheep BES [16].

In a non-model, non-domestic species such as the tammar wallaby, there are insufficient BES available for the creation of a consensus alignment, and no closely related species with which to align. However, there are considerable gene mapping data available from long-running efforts to produce a linkage map and a FISH-based physical map of this species [17,18], although there is no RH map. We therefore used all the available tammar wallaby mapping data to create an integrated map, using the Location Database (LDB) tool.

LDB is a bioinformatics tool first created to integrate all available human maps into a single map before the human sequence assembly was available [19]. Inputs can include linkage maps (in cM), RH maps (in cR) and fluorescent *in situ *hybridisation (FISH) data (cytogenetic locations). The output is a single map comprising the loci from all of the input data. Each locus is allocated a cM, cR and cytological band location, even if it has actually been mapped using only one of these methods. Most importantly, the position of each locus on a chromosome in the genome of that species is estimated in kb, just as in an actual sequence assembly. This map-integration strategy has been shown to be very powerful and accurate, and has been applied to livestock species not then sequenced [20].

For the tammar wallaby, there are cytogenetic and linkage-mapping resources that can be integrated to provide a framework for the genome assembly. A linkage map has been under construction for many years: the first-generation map was published in 2002, incorporating 64 loci on all autosomes and the X chromosome [17]. Since then, many loci have been added, creating a second-generation linkage map comprising 148 loci, with a total size of 1402.4 cM, and an average inter-locus distance of 10.9 cM (Wang et al., unpublished data). The chromosomes of the tammar wallaby have been thoroughly characterized [18], and have been mapped by FISH using BACs containing functional loci with human orthologues. The strategy of screening for tammar orthologues of genes that flank regions with a conserved gene content in both humans and opossum (regions of conserved synteny) [21] has delivered the locations of 492 genes in the tammar wallaby, including nearly all the new loci in the second-generation linkage map [22] (Deakin et al., unpublished data). The initial focus in the present paper is therefore to integrate all available tammar wallaby mapping information to provide the "backbone" for a tammar map that is based only on tammar wallaby information.

The integration of these hard-won tammar wallaby resources produces a map that is valuable, but not sufficiently complete or high-resolution to be adequate for genome assembly, e.g. some of the FISH locations on small chromosome arms cannot be specified to a greater precision than a whole arm. Comparative mapping information can be utilised to refine the relatively crude FISH locations of some tammar wallaby genes. Thus, without compromising the "backbone" of the integrated map that is based on tammar wallaby linkage and cytogenetic maps, comparative information from the most closely related sequenced genome is used to fine-tune the location of genes in the integrated map for which only crude tammar locations are available. Finally, a tammar wallaby virtual genome map is created by assuming conservation of synteny in the intervals between genes in the integrated map, again using primarily information from the tammar wallaby's nearest sequenced evolutionary neighbour, opossum, and (where this information is insufficient) from human. Although this latter step provides only a null hypothesis of the actual order and relative location of genes not yet mapped in the tammar wallaby, in the absence of any more direct information, this strategy has produced a virtual map that can be used as the framework for the initial assembly of the tammar wallaby genome sequence.

The aims of this paper, therefore, are (a) to identify blocks of conserved synteny and hence evolutionary breakpoints in the tammar wallaby genome; (b) to create a first-generation integrated tammar wallaby map by combining all available tammar mapping information for genes that have been mapped in tammar wallaby, making use of orthologous marker locations in opossum and human to fine tune loci FISH-mapped in tammar wallaby with low resolution; and (c) to create a first-generation tammar wallaby virtual genome map by utilising comparative opossum and human mapping information, for genes that have not been mapped in tammar wallaby.

## Results

The integrated and virtual maps of the tammar wallaby genome were created from the second-generation linkage map, comprising 148 loci [22], together with FISH-mapping data for 492 loci (Deakin et al., unpublished data), using the strategy described by Liao et al [20]. Firstly, we estimated the physical size and linkage size of each tammar wallaby chromosome, and the total genome size. We then identified blocks of conserved synteny between tammar wallaby and opossum (or human when no clear conserved syntenic blocks between tammar wallaby and opossum could be observed), and identified breakpoints between them. Next we estimated the size of each chromosome arm, and estimated band locations in kb by linear interpolation. We then created files of FISH locations, linkage-map locations and breakpoints for each chromosome, and also an orthologue-location file for FISH-mapped markers, all of which were used to create the integrated map. Finally, orthologue-location files were created for all the conserved syntenic blocks, and these were used to create the virtual genome map. Chromosome nomenclature follows the usual practice: HSA (human), MEU (tammar wallaby) and MDO (opossum).

### Step 1: Estimation of Mb and cM size of each chromosome

The tammar wallaby genome consists of seven pairs of autosomes and one pair of sex chromosomes, X and Y. The physical size (in Mb) of each tammar wallaby chromosome was determined by direct comparison with the well established sizes of human chromosomes. The direct comparison was performed by bivariate flow karyotyping and the results are presented in Table 1. Tammar wallaby chromosomes 1-6 are all larger than human chromosomes. For instance, MEU1 is about twice the size of HSA1. Notable is the size of MEUX of 150 Mb, which is a value between HSA8 and HSAX. Despite MEUX having a much smaller gene content than HSAX and being regarded as the ancestral therian X [21], its DNA content is comparable with HSAX, due to the addition of repetitive sequences of this NOR-bearing chromosome. The estimated total linkage length for each chromosome, also shown in Table 1, was estimated as its cM length in the second-generation linkage map, divided by the estimated proportion of chromosome covered by that linkage map (Wang et al. unpublished).

**Table 1:** Chromosomes sizes and genome size (Mb and cM).

Tammar wallaby chromosome (MEU)	Size (Mb)	Size (cM)
1	486	342.58
2	367	246.98
3	355	242.39
4	340	185.76
5	340	232.68
6	286	134.18
7	133	123.78
X	150	189.75^a^

Total	2457	1698.10

^a ^189.75 cM is the estimated cM length of Xq only because there is a large NOR in Xp which precludes a reasonable estimate of the total cM length of the X chromosome.

### Step 2: Estimation of genome size

The tammar wallaby genome size is estimated to be 2.457 Gb and 1698.10 cM determined by adding up the chromosome sizes given in Table 1. This physical size is smaller than the human genome size of 3.08 Gb [23].

### Step 3: Identification of blocks of conserved synteny and evolutionary breakpoints between tammar wallaby, opossum and human

**Figure 1:**
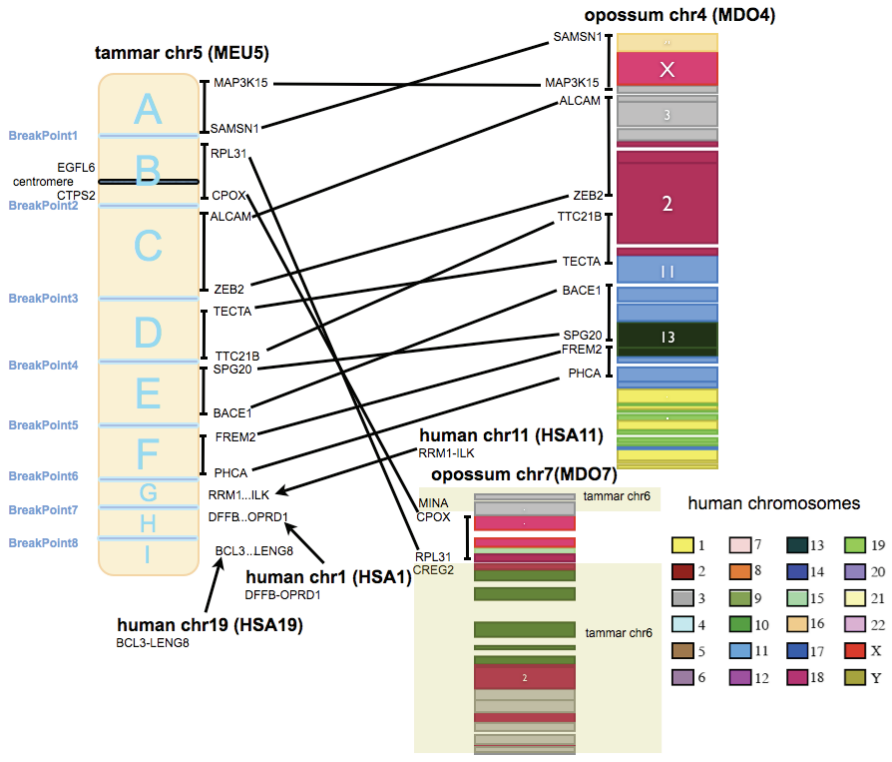
**Comparative map between tammar chromosome MEU5, and corresponding opossum (MDO) and human (HSA) chromosomes**. The different coloured regions in MDO4 and MDO7 indicate blocks of opossum-human conserved synteny (with the largest human chromosome blocks identified in the figure, and all relevant human chromosomes indicated in the colour-scheme box). Letters A to I indicate blocks of tammar-opossum/human conserved synteny, with each of the eight breakpoints (and the orientation of each block) indicated by flanking markers. The two markers to the left of MEU5 flank the centromere identified by FISH mapping. The sections of MDO7 shadowed as MEU6 indicate that all those sections of MDO7 except the block conserved with MEU5, are conserved with MEU6.

For every gene FISH-mapped in the tammar wallaby, the location of its orthologue in the opossum and human genomes was determined. Blocks with conserved gene content were identified, as were genes that marked the end of each block and hence evolutionary breakpoints. Estimates were obtained of the size (in opossum or human in some cases) of each block of conserved synteny, and sizes of the blocks were summed over chromosomes and scaled to the estimated total size of the relevant tammar wallaby chromosome, creating a location (in kb) in tammar wallaby for each breakpoint. These blocks were defined to reflect the FISH-mapping results for all markers. Orientation of each block on the tammar wallaby chromosome was deduced from their FISH-mapping data wherever possible. Amongst the total of 84 conserved syntenic blocks, there were 23 with only one orthologous gene, 19 of which are on MEU3. When FISH data did not suggest a clear orientation of the block and for the one-probe blocks, the gene orders were retained the same as in the reference genome (opossum or human). With the one-probe blocks comprising only 8 Mb, compared with 58 Mb coverage of multi-gene blocks, this assumption concerning gene order was not critically important. A summary of the estimated breakpoints for all autosomes is presented in Table 2. The X chromosome was excluded from this table because, even though all the loci FISH-mapped to MEUX are located on MDOX, there were no obvious regions of conserved synteny, so it was not possible to estimate the breakpoints. Figure 1 illustrates the blocks of conserved synteny, and the breakpoints, for a typical tammar wallaby chromosome, MEU5. Similar illustrations for each of the other tammar wallaby chromosomes are presented in Additional File 1, Figure S1, Additional File 2, Figure S2, Additional File 3, Figure S3, Additional File 4, Figure S4, Additional File 5, Figure S5, and Additional File 6, Figure S6.

**Table 2:** Breakpoints between blocks of conserved synteny (with respect to opossum or human [in italics]) in tammar wallaby autosomes.

	Breakpoint location (Mb) in Tammar wallaby autosome (MEU)						
**Breakpoint No**.	**MEU1**	**MEU2**	**MEU3**	**MEU4**	**MEU5**	**MEU6**	**MEU7**

1	10.457	44.000	6.975	24.970	37.729	137.164	68.960
2	18.164	66.963	12.959	59.325	71.500	148.722	-
3	49.991	98.799	13.762	77.755	152.272	159.539	-
4	74.210	220.170	22.684	86.055	206.066	188.129	-
5	90.479	252.521	25.237	93.325	267.109	209.119	-
6	124.826	266.054	26.008	109.093	291.421	221.964	-
7	153.320	-	29.018	139.119	*293.771*	238.312	-
8	165.519	-	64.847	280.434	*324.806*	283.660	-
9	191.563	-	66.291	281.743	-	-	-
10	207.073	-	72.584	309.767	-	-	-
11	230.327	-	79.807	-	-	-	-
12	234.147	-	102.372	-	-	-	-
13	236.706	-	107.559	-	-	-	-
14	318.217	-	113.096	-	-	-	-
15	417.080	-	120.422	-	-	-	-
16	418.331	-	129.009	-	-	-	-
17	455.644	-	137.739	-	-	-	-
18	-	-	140.468	-	-	-	-
19	-	-	141.190	-	-	-	-
20	-	-	141.992	-	-	-	-
21	-	-	*156.758*	-	-	-	-
22	-	-	*170.135*	-	-	-	-
23	-	-	*175.506*	-	-	-	-
24	-	-	*180.577*	-	-	-	-
25	-	-	*187.518*	-	-	-	-
26	-	-	*210.810*	-	-	-	-
27	-	-	*211.725*	-	-	-	-

As an example of the approach, MEU5 comprises nine blocks of conserved synteny, indicated as blocks A-I in Figure 1, with their boundaries defined by eight breakpoints. Loci on the right-hand side of MEU5 are end markers for each conserved syntenic block, which were FISH-mapped in tammar wallaby. The last three blocks (G, H and I) have no clear opossum counterparts, but show marked conserved synteny with regions of HSA 11, 1 and 19, respectively. However, their FISH-mapping locations in tammar wallaby are too close to resolve, so we could not determine the orientation of these blocks in tammar wallaby. Different tammar wallaby chromosomes showed different degrees of evolutionary rearrangement, the number of blocks of conserved synteny ranging from 2 (MEU7) to 28 (MEU3), with an average of 12.

The only difficulty encountered in identifying blocks of conserved synteny in autosomes concerned the p arm of MEU2 (Additional File S2), for which only approximately 6 Mb (out of a total estimated length for 2p of 44 Mb) could be identified as being orthologous to human or opossum chromosomal segments (HSA11 or MDO5 and MDO8). Therefore, breakpoint analyses were not carried out on MEU2p.

### Step 4: Estimation of centromere position and arm lengths

In all tammar wallaby chromosomes except MEU5, the FISH-mapping data from tammar wallaby indicated that the centromere was located between two blocks of conserved synteny. Thus it was placed at the breakpoint between those two blocks. For MEU5, the FISH-mapping data suggested the centromere is located within a conserved block, between markers *EGFL6 *and *CTPS2*. The location of this centromere was first estimated as the mid-point (in the opossum assembly) between these two markers. The distances between each of these markers and the two breakpoints defining this conserved block in opossum were then rescaled onto the tammar wallaby version of this conserved block, to provide an estimate of the location of this centromere in the tammar wallaby.

The size of each arm of each autosome was then estimated in Mb from the chromosome sizes from Step 1 and the relative conserved syntenic block sizes on each side of a centromere. The arm sizes in cM were firstly calculated using the same p and q arm ratio as in Mb. Where necessary, they were then minimally adjusted to ensure that the centromere was located between two flanking markers, one of which had been FISH-mapped to the p arm and the other to the q arm. Table 3 shows the estimated size of each autosome arm. No orthologous regions were identified in the short arm of MEU2, so arm sizes in Mb and cM for MEU2 p and q were estimated from the arm ratio of 12:88 in the published tammar wallaby karyotype [17]. An arm file with the p and q arm Mb and cM lengths was then created for each chromosome.

**Table 3:** Arm size estimated for each autosome.

Chromosome	Arm	Arm size (Mb)	Arm size (cM)
1	p	90.479	65.00
1	q	395.521	277.58
2	p	44.00	29.64
2	q	323.00	217.34
3	p	141.992	94.00
3	q	213.008	148.39
4	p	109.093	68.73
4	q	230.907	117.03
5	p	66.812	53.52
5	q	273.188	179.17
6	p	137.164	64.35
6	q	148.836	69.83
7	p	68.960	60.65
7	q	64.040	63.13

### Step 5: Estimation of the kb location of the beginning and end of each band in the idiogram

The proportional location of each border of each band was estimated from the standard idiogram [18] and translated into kb locations by linear interpolation within the total kb size of the relevant chromosome arm, as determined in step 4. A band file was created for each chromosome and, after an iterative curation process as described in the Methods section, the final band definitions was determined, as presented in Additional File 7, table S7.

### Step 6: Creation of an input file from each source of mapping data

Linkage map and FISH-map input files were created for each chromosome, as described in the Methods section. A FISH-orthologue file was created for each block of conserved synteny with either opossum (preferably) or human, to enable the fine-tuning of the location of FISH-mapped markers that would otherwise be located via the LDB process in the centre of their band(s). A breakpoints file was also created for each chromosome.

### Step 7: Creation of an integrated map

For each chromosome, the integration process was run using the LDB software [20], with the integration order being FISH-map file, linkage-map file, breakpoints file and FISH-orthologue file(s), followed by manual curation of the band definitions by checking the discrepancies between the LDB predicted and the FISH-mapped band locations, as described in the Methods section, and running the integration process again until no further discrepancies existed. This produced an integrated map for each chromosome, combining all available FISH-mapping and linkage-mapping data. For markers whose only tammar wallaby data comprised FISH locations, their predicted locations in the integrated map were fine-tuned by the comparative information contained in the FISH-orthologue files.

As an example, portions of the integrated map of MEU5 are shown in Table 4. The map extends from the 5p telomere (5ptr) at 0 kb to the 5q telomere (5qtr) at 340000 kb. The 5qtr location corresponds to the size of chromosome 5 (Table 1). Similarly, the predicted locations in the linkage map of MEU5 range from 0 cM to 232.68 cM, the latter location corresponding to the estimated cM length of MEU5 (Table 1). Included in Table 4 are each of the eight breakpoints in this chromosome, together with loci flanking these breakpoints and the loci adjacent to the telomeres. Complete integrated maps for all MEU autosomes are presented in Additional File 8, table S8, and are also available through the Comparative Location Database [22]. In total, there are 553 markers in the first-generation integrated map across all autosomes.

**Table 4:** Portions of the integrated map for tammar wallaby chromosome 5 (MEU5).

Marker (Human gene symbol)	Location (kb)	Location (cM)	Location (band)
ptr	0	0.00	p3
**MAP3K15**	52	0.04	p3
. . . *			
**CXADR**	36862	29.53	p2
**SAMSN1**	37623	30.14	p2
Breakpoint 1	37729	30.22	p2
RPL31	37869	30.34	p2
. . .			
EGFL6	65104	52.15	p1
**cen**	66812	53.52	cen
**CTPS2**	68521	54.64	q1
. . .			
CPOX	71284	56.45	q1
Breakpoint 2	71500	56.59	q1
ALCAM	74180	58.35	q1
. . .			
ZEB2	141002	102.18	q2
BREAKPOINT3	152272	109.57	q2
TECTA	154477	111.01	q2
. . .			
KJW192	202612	142.58	q2
BREAKPOINT4	206066	144.85	q2
SPG20	207288	145.65	q2
. . .			
BACE1	264904	183.44	q2
BREAKPOINT5	267109	184.88	q2
HPX	268326	185.68	q2
. . .			
PHCA	287618	198.34	q2
BREAKPOINT6	291421	200.83	q2
CCKBR	292596	201.60	q2
. . .			
RRM1	292596	201.60	q3
**BREAKPOINT7**	293771	202.37	q3
**DFFB**	294925	203.13	q3
. . .			
LEPRE1	324670	222.64	q3
BREAKPOINT8	324806	222.73	q3
BCL3	332209	227.58	q3
...			
**LENG8**	339572	232.41	q3
qtr	340000	232.68	qtr

Markers in bold are also shown in the virtual genome map (Table 5)

* "..." rows indicate where there are markers not listed in the table.

### Step 8: Creation of a virtual genome map

Opossum and human orthologue files, containing opossum (wherever possible) or human orthologous locations for all mapped and unmapped genes in each conserved syntenic block, were constructed for each block of conserved synteny, as described in the Methods section. A virtual genome map for each chromosome was then constructed by incorporating the relevant orthologue files into the integrated map for that chromosome, using the same LDB software.

The virtual genome map for a tammar wallaby chromosome therefore comprises all genes whose location on that chromosome is supported by available tammar wallaby and orthologous information, including the many genes that have not yet been mapped in this species. In this virtual genome map, three confidence levels are defined. The highest (confidence level 2) is assigned to loci that have been mapped (linkage and/or FISH) in the tammar wallaby. The middle level (confidence level 1) is applied to those loci not mapped in the tammar wallaby but which occur in a block of conserved synteny whose orientation in tammar wallaby can be deduced from FISH-mapping data. The lowest level (confidence level 0) is given to those loci, which are neither mapped in tammar wallaby, nor have any evidence from tammar wallaby supporting their order within their conserved syntenic blocks.

To illustrate this outcome, portions of the virtual genome map for MEU5 are presented in Table 5. The total length of the chromosome is the same as in the integrated map, namely 340000 kb and 232.68 cM. It can be seen in Table 5 that two opossum markers (indicated by the prefix ENSMODG) have been interpolated between *CXADR *and *SAMSN1*. Similarly, 13 opossum markers have been interpolated between the centromere and *CTPS2*. These two sets of interpolated opossum markers have a confidence level of 1, indicating that although they have not been mapped in tammar wallaby, in each case their orientation could be deduced from the known orientation of the two flanking markers that are also in that group in the opossum assembly (*CXADR *and *SAMSN1 *for the first group, and *cen *and *CTPS2 *for the second group). Next, seventeen human markers (indicated by the prefix ENSG) have been interpolated between Breakpoint 7 and, *DFFB *and four human markers between *LENG8 *and *qtr*. These two sets of markers have a confidence level of 0, indicating that although they are predicted (from comparative mapping data) to be located between the relevant markers shown in bold, their orientation and order are not known with any certainty.

**Table 5:** Portions of the virtual genome map for tammar wallaby chromosome 5 (MEU5).

Marker (Human gene symbol)	Marker (Ensembl ID)^a^	Location (kb)	Confidence Level^b^	Location (cM)	Location (band)
ptr		0	NA	0.00	p3
**MAP3K15**	ENSMODG00000009020	52	2	0.04	p3
. . . *					
**CXADR**	ENSMODG00000020956	36862	2	29.53	p2
USP25	ENSMODG00000020954	37113	1	29.73	p2
NRIP1	ENSMODG00000020951	37474	1	30.02	p2
**SAMSN1**	ENSMODG00000020950	37623	2	30.14	p2
. . .					
**cen**		66812	NA	53.52	cen
ASB9	ENSMODG00000017264	66989	1	53.64	q1
ASB11	ENSMODG00000017240	67067	1	53.69	q1
PIGA	ENSMODG00000017236	67096	1	53.71	q1
FIGF	ENSMODG00000017234	67131	1	53.73	q1
PIR	ENSMODG00000017232	67233	1	53.80	q1
BMX	ENSMODG00000017228	67298	1	53.84	q1
ACE2	ENSMODG00000017222	67358	1	53.88	q1
TMEM27	ENSMODG00000017212	67469	1	53.95	q1
CA5B	ENSMODG00000017209	67537	1	54.00	q1
ZRSR2	ENSMODG00000017202	67566	1	54.01	q1
AP1S2	ENSMODG00000017197	67589	1	54.03	q1
GRPR	ENSMODG00000017191	68033	1	54.32	q1
S100G	ENSMODG00000017180	68470	1	54.61	q1
**CTPS2**	ENSMODG00000017176	68521	2	54.64	q1
. . .					
**BREAKPOINT7**		293771	NA	202.37	q3
MORN1	ENSG00000116151	293771	0	202.37	q3
RER1	ENSG00000157916	293824	0	202.41	q3
PEX10	ENSG00000157911	293834	0	202.41	q3
PLCH2	ENSG00000149527	293889	0	202.45	q3
PANK4	ENSG00000157881	293913	0	202.46	q3
HES5	ENSG00000197921	293928	0	202.47	q3
C1orf93	ENSG00000157870	293972	0	202.50	q3
MMEL1	ENSG00000142606	293975	0	202.50	q3
AL831784.17	ENSG00000215913	294014	0	202.53	q3
ACTRT2	ENSG00000169717	294291	0	202.71	q3
PRDM16	ENSG00000142611	294327	0	202.74	q3
TPRG1L	ENSG00000158109	294748	0	203.01	q3
WDR8	ENSG00000116213	294753	0	203.01	q3
TP73	ENSG00000078900	294769	0	203.03	q3
CCDC27	ENSG00000162592	294845	0	203.08	q3
LRRC47	ENSG00000130764	294866	0	203.09	q3
KIAA0562	ENSG00000116198	294892	0	203.11	q3
**DFFB**	ENSG00000169598	294925	2	203.13	q3
...					
**LENG8**	ENSG00000167615	339572	2	232.41	q3
LENG9	ENSG00000182909	339581	0	232.42	q3
LAIR2	ENSG00000167618	339612	0	232.44	q3
FCAR	ENSG00000186431	339894	0	232.62	q3
GP6	ENSG00000088053	340000	0	232.68	qtr
qtr		340000	NA	232.68	qtr

Markers in bold are also shown in the integrated map (Table 4)

* "..." rows indicate where there are markers not listed in the table.

^a^. ENSMODG = opossum; ENSG = human

^b^. 2 = high: linkage and/or FISH-mapped tammar wallaby markers; 1 = medium: not mapped in the tammar wallaby but occurs in a block whose orientation could be deduced from tammar wallaby FISH-mapping data; 0 = low: neither mapped in tammar wallaby nor has any tammar wallaby evidence supporting an order.

Across all autosomes, the virtual genome map comprises 14336 loci and has a size of 2457 Mb. Because of its size, the entire tammar wallaby virtual genome map could not be included in the Additional Files. It is available through the Comparative Location Database [22].

## Discussion

Given the large quantity of data incorporated, the first-generation integrated and virtual genome maps reported here will enhance significantly genome research in the tammar wallaby (a valuable model kangaroo species), and facilitate the assembly of the genome sequence of this species.

Whenever comparative data were required, we have been conservative in using wherever possible the genome of the most closely related sequenced marsupial. The tammar wallaby and the opossum diverged around 70 mya [11], comparable with the divergence within eutheria, amongst which much use has been made of comparative information, e.g. dog and human [24]; sheep and human [16]. The next-best choices are eutherians, which are more than twice as distant (diverging 150 mya) [9,10]. These realities provide a strong justification for the present strategy of drawing comparative information from opossum in preference to eutherians.

In addition, the remarkable conservation of chromosome arrangement in marsupials makes this approach particularly appropriate for the tammar wallaby. Whereas the eutherian genome has been grossly rearranged in many lineages, there is very strong conservation of synteny between tammar wallaby and opossum [25], even to the extent of whole chromosome arms being conserved [26]. When the integrated map was used to create the virtual genome map, the preferred strategy was, once again, to rely as much as possible on the tammar wallaby's nearest sequenced evolutionary neighbour, and then to turn to one of the most mature genome assemblies, namely human, only in the minority of cases where the opossum information was not sufficient.

Of course there will be errors in the order and relative location of loci, especially in the virtual genome map: the integration of data from conserved blocks of synteny means that the location of most tammar wallaby genes in the virtual genome map is predicted on the basis of their order in other species. However, as argued above, in using whenever possible comparative mapping data from opossum, we are, in effect, relying on better comparative information then has been the basis of the utilisation of comparative mapping information within eutherians.

Estimation of conserved-synteny blocks is not a simple process, and errors are certain to have been made, given the relative paucity of information available in the tammar wallaby. For the markers with the lowest confidence level, it is important to note that close-range locus order presented is just one of several equally likely possibilities.

As more sequence-level comparative data become available, these blocks will be better defined.

In principle, the overall aim of creating an integrated map is to combine together in a rational manner all available mapping information in the species of interest, without recourse to any information from other species. In the creation of the integrated map of sheep, for example [20], comparative data were used only in the local repositioning of loci that had all been FISH-mapped to the same chromosomal band. As discussed by Liao et al. [20], this did not compromise the essential integrity of the integrated map in reflecting all available sheep data: it simply provided a first estimate of the order of a set of loci that are known to be located within a particular band. In the case of the tammar wallaby, there was a lack of orthologues mapped with sufficient resolution in this species, which precluded the local ordering of loci that had been FISH-mapped to a particular band. The best solution, given the lack of resources to create a denser physical map, was to estimate evolutionary breakpoints in the tammar wallaby with respect to the opossum (wherever possible) and human assemblies. Whilst this provides an additional compromise to the integrity of the integrated map, this does not alter any mapping data gleaned from the wallaby alone. Therefore, in practice, it does not provide any additional compromise to the integrity of the wallaby-mapping data.

How does this first-generation virtual genome map compare with the resources used in genome assemblies in other species? The bovine genome sequence [27] was assembled onto a single RH map [28]. The opossum genome assembly [5] was assigned to chromosomes based primarily on FISH-mapping of BACs from scaffolds [29], with support from the second of two linkage maps whose terminal markers had also been FISH-mapped [30]. Our tammar wallaby first-generation virtual genome map is more comprehensive than either of these strategies, since it is based on all available mapping information from the species itself, combined in a rational manner, supplemented by comparative mapping data. This integrated map is better and more useful than either of its components considered alone. Obviously it would be desirable to obtain more mapping information (both linkage and physical) for the tammar wallaby. As such data become available in the future, they will be used in the construction of second-generation integrated and virtual genome maps. In the meantime, the maps described in this paper are the best available at this time; they utilize all available information to create the most complete maps of the tammar wallaby chromosomes that can be produced at this time.

As pointed out by Lewin et al. [31], "Every genome sequence needs a good map". Genome sequence itself is not sufficient to enable a chromosome assembly or construction of good comparative maps to reveal hidden evolutionary stories. Good genome maps (e.g. physical maps, RH maps, linkage maps) are a necessary complement to genome sequence. However, they are of limited use in isolation. What is needed is a means of integrating all available mapping data for a species into a single map. The first-generation integrated map reported in this paper achieves this aim for the tammar wallaby, and has enabled the creation of a first-generation virtual genome map for this species, combining the integrated map with comparative mapping data from species with more mature chromosome assemblies.

By combining the first-generation virtual genome map presented in this paper with the Ensembl annotation [13] of the initial (Meug_1.0) tammar wallaby assembly, it is now possible to construct the first draft chromosome assembly for the tammar wallaby. In their annotation process, Ensembl were able to create 10257 "gene-scaffolds" comprising two or more Meug_1.0 scaffolds. Of these, 7027 have one gene in common with the virtual genome map, and an additional 953 have more than one gene in common with the virtual map, giving a total of 7980 gene-scaffolds that can be incorporated into a chromosome assembly, based on the virtual genome map. Thus 78% of the Ensembl gene-scaffolds can be incorporated into a tammar wallaby chromosome assembly, and 9% of the gene-scaffolds can be orientated in this chromosome assembly.

In addition to the Ensembl gene-scaffolds, there are another 1175 Meug_1.0 scaffolds that have at least one gene in common with the virtual genome map, and 54 Meug_1.0 scaffolds that have multiple genes in common with the virtual genome map. The total size of gene-scaffolds and scaffolds that can be incorporated into a chromosome assembly is 533,684,520 bp, which is 22% of the estimated tammar wallaby genome size (2457 Mb). This chromosome assembly includes 10522 of the 15290 protein-coding genes identified in the Ensembl annotation. In other words, the virtual genome map enables the creation of a chromosome-based tammar wallaby genome assembly that includes a high proportion (69%) of protein-coding genes identified in the sequence data. This compares with the few gene-scaffolds whose location can be determined solely from the integrated map built almost exclusively from tammar wallaby mapping information: only 265 gene-scaffolds have one gene in common with the integrated map and three gene-scaffolds have more than one gene in common with the integrated map. Also the virtual genome map has been tested in the recent tammar wallaby genome sequence assembly attempt and has significantly enhanced the N50 of the assembly [32].

## Conclusions

Construction of a tammar wallaby first-generation integrated map has allowed prediction of the genomic content and organization of the wallaby genome via a first-generation virtual genome map, which will be useful as a resource for assembly of the wallaby genome sequence. Since marsupial chromosomes are highly conserved [25,26,33], the results from this study, when combined with the opossum assembly, will inform studies on the genome of other marsupial species. In particular, the detailed breakpoints between opossum/human and tammar wallaby revealed in this study will be useful for studies of genome evolution in marsupials, construction of an ancestral marsupial karyotype, and comparisons with the genomes of eutherians, and with non-mammal vertebrates.

## Methods

### Estimating chromosome and genome size by flow cytometry

Tammar wallaby chromosome sizes were determined by bivariate flow karyotyping according to Trask et al. [34], Boschman et al. [35], Langford et al. [36]. Chromosome preparations of tammar wallaby and human were measured together and separately but sequentially with the same flow cytometry settings, allowing a direct comparison between the tammar wallaby chromosomes and the human chromosomes. Following the protocols described in the above reports, chromosomes were isolated from mitotic cells obtained by blocking with colcemid. The chromosomes were stained with Hoechst 33258 and chromomycin A3, which have a preferential binding to AT- and GC-rich DNA, respectively. The "DNA-line" in the flow karyotypes was drawn from the origin through human chromosome 4. Each human and tammar wallaby chromosome peak was projected onto this line, and the distance from the origin to this projection was an estimate of the DNA content of that particular chromosome. The established human chromosome sizes (Ensembl) were used as references for the tammar wallaby chromosomes taking into account both conversion and offset. Tammar wallaby genome size was determined by adding up the chromosome sizes.

### Breakpoint analysis: estimation of blocks of conserved synteny and evolutionary breakpoints between tammar wallaby, opossum and human

For each of the 492 genes FISH-mapped in the tammar wallaby, the location of its orthologue in the opossum and human genomes was determined. Blocks of conserved synteny were identified. Genes that mark the end of each block were noted. These genes indicate evolutionary breakpoints. The size of each block of conserved synteny was estimated in opossum or, in the few cases where this was not possible, in human. For each tammar wallaby chromosome, the total size of all blocks of conserved synteny that comprise that chromosome was calculated. This total was then scaled to the estimated total size of the relevant tammar wallaby chromosome, creating a location (in kb) in tammar wallaby for each breakpoint.

### Creation of input files and their use in creating the integrated map

An arm file and a band file were created for each chromosome. Each arm file comprises four columns: the first column identifies the chromosome arm (i.e. p or q), the second column is the arm size in Mb, then cM length in male and cM length in female. To combine the available linkage data with the maximum number of markers in the present context, the sex-pooled cM size of the arm was used in the last two columns. Each band file also has four columns, namely the band name (e.g. p1, q2), band size in Mb, band start location in Mb, and band end location in Mb.

A FISH-map file for each chromosome comprised three columns for each FISH-mapped locus, namely locus name, and then two fields indicating the ID of the band or bands to which that gene has been FISH-mapped. If the gene had been FISH-mapped to just a single band, then the second and third columns both have the ID of that band. If the gene had been mapped to a region encompassing two or more bands, the second column contained the ID of the band at the left (p telomere side) end of that region, and the third column contained the ID of the band at the right (q telomere side) end of that region.

For each chromosome, a linkage-map file comprised three columns, namely locus name, male cM location and female cM location. Similar to the arm file, the sex-pooled cM location of each locus was inserted in each of the second and third columns of the linkage-map file. The cM locations entered in the file are slightly different from those in the second-generation linkage map [22], having been scaled to correspond to the full-coverage map lengths shown in Table 1 by adding an offset value to each marker location corresponding to the cM equivalent of the p-telomeric end of the chromosome not covered by the second-generation linkage map. To calculate this for each chromosome, we used the estimate of the size of the uncovered p-telomere end of the chromosome as a percentage of the size of the chromosome covered by the second-generation linkage map, as calculated for the second-generation linkage map (Wang et al, unpublished data), where it is called m%. The offset value for a chromosome was then calculated by multiplying the m% by the length of the second-generation linkage map. The offset values for MEU1 to MEU7 were 12.21, 29.10, 7.33, 16.34, 3.60, 3.53 and 1.49 respectively. In addition to actual loci, the linkage-map file also contained rows for the p telomere, the q telomere and the centromere.

A breakpoints file was also created for each chromosome, comprising two columns, the first being breakpoint ID and the second being breakpoint location in kb (identified in the breakpoint analysis). These files also contained rows for both telomeres and the centromere.

A FISH-orthologue file was also created for each block of conserved synteny with either opossum (preferably) or human, as gleaned from Ensembl. These FISH-orthologue files enable the fine-tuning of the location of FISH-mapped markers that would otherwise remain located, via the LDB process, in the centre of their band(s). The three columns in each FISH-orthologue file were name of locus FISH-mapped in tammar wallaby, kb location of the orthologue of that locus in opossum/human, and opossum/human chromosome name. To enable correct integration, each FISH-orthologue file also contained relevant breakpoints, the p telomere, the q telomere and the centromere.

The integrated map was built with LDB software and the above input files. This map creation process involved initial running of the LDB software, manual curation of the band definitions by minimally extending some band boundaries to include all FISH-mapped markers where these fell outside the LDB predicted band positions, and repeating this process until no further discrepancies existed between the predicted and FISH mapped band locations.

### Creating the virtual genome map

The construction of the virtual genome map required the creation of one more type of input file, namely orthologue files. Orthologue files contain opossum or human orthologous locations for all mapped and unmapped genes in each conserved syntenic block. Prior to the construction of orthologue files, the list of loci in each conserved block was compared between opossum and human. For those blocks that were clearly visible in both species, a very small number of loci within those blocks had locations that were not consistent with the block. These so-called "orphans" were excluded from the blocks prior to creation of the orthologue files. An opossum or a human orthologue file was thus constructed for each block of conserved synteny. Each file comprised three columns, namely locus ID (in this case the Ensembl ID of the opossum or human locus), the bp location of that locus in opossum or human, and the opossum or human chromosome on which that locus resides. In addition, rows were inserted for the two telomeres, the centromere, and the breakpoints for that conserved block.

## Abbreviations

BAC: bacterial artificial chromosome; BES: BAC-end sequences; cM: centimorgan; cR: centiRay; FISH: fluorescence in-situ hybridization; HAS: human (*Homo sapiens*); kb: kilobase; LDB: Location DataBase; Mb: megabase; MDO: opossum (*Monodelphis domestica*); MEU: tammar wallaby (*Macropus eugenii*); pg: picogram; RH: radiation hybrid; SNP: single nucleotide polymorphism.

## Authors' contributions

CW wrote the first draft of the manuscript. CW and FWN drafted subsequent versions, with substantial input from JAMG and JED. CW constructed the integrated and virtual maps under FWN's close supervision. JED gave extensive feedback and suggestions on the maps, which greatly helped the development of additional strategies required for the creation of the maps. WR calculated genome and chromosome sizes using flowcytometry data. KB provided formal supervision in the latter stages of CW's postgraduate candidature, and provided very useful feedback on the manuscript. JAMG and KRZ provided the initial impetus for this project. JAMG provided financial and laboratory support. KRZ provided close supervision in the early stages of the project. All authors read and approved the final manuscript

## Supplementary Material

Additional file 1 **Figure S1**. Comparative map between tammar chromosome 1 (MEU1) and opossum chromosomes 1 and 6 (MDO1, MDO6). Note: For easier comparison with MEU1, MDO6 is shown with its q telomere at the top and its p telomere at the bottom. Click here for file

Additional file 2 **Figure S2**. Comparative map between tammar wallaby chromosome 2 (MEU2) and opossum chromosome 2 (MDO2). Note: For easier comparison with MEU2, MDO2 is shown with its q telomere at the top and its p telomere at the bottom. Click here for file

Additional file 3 **Figure S3**. Comparative map between tammar wallaby chromosome 3 (MEU3) and opossum/human chromosomes (MDO6, MDO8, HSA12, HSA22). Note: For easier comparison with MEU3, MDO6 is shown with its q telomere at the top and its p telomere at the bottom. Click here for file

Additional file 4 **Figure S4**. Comparative map between tammar wallaby chromosome 4 (MEU4) and opossum chromosome 3 (MDO3). Click here for file

Additional file 5 **Figure S5**. Comparative map between tammar wallaby chromosome 6 (MEU6) and opossum chromosomes 5 and 7 (MDO5, MDO7). Click here for file

Additional file 6 **Figure S6**. Comparative map between tammar wallaby chromosome 7 (MEU7) and opossum chromosomes 1 and 5 (MDO1, MDO5). Click here for file

Additional file 7 **Table S7**. Band location estimated for each autosome. Click here for file

Additional file 8 **Table S8**. The tammar wallaby integrated map. Click here for file
