# Supplementary figures and images for: A first-generation integrated tammar wallaby map and its use in creating a tammar wallaby first-generation virtual genome map
Source: BMC Genomics. 2011 Aug 19;12:422. doi: 10.1186/1471-2164-12-422 (PMC3170641; doi:10.1186/1471-2164-12-422)

tammar chr2

opossum chr2

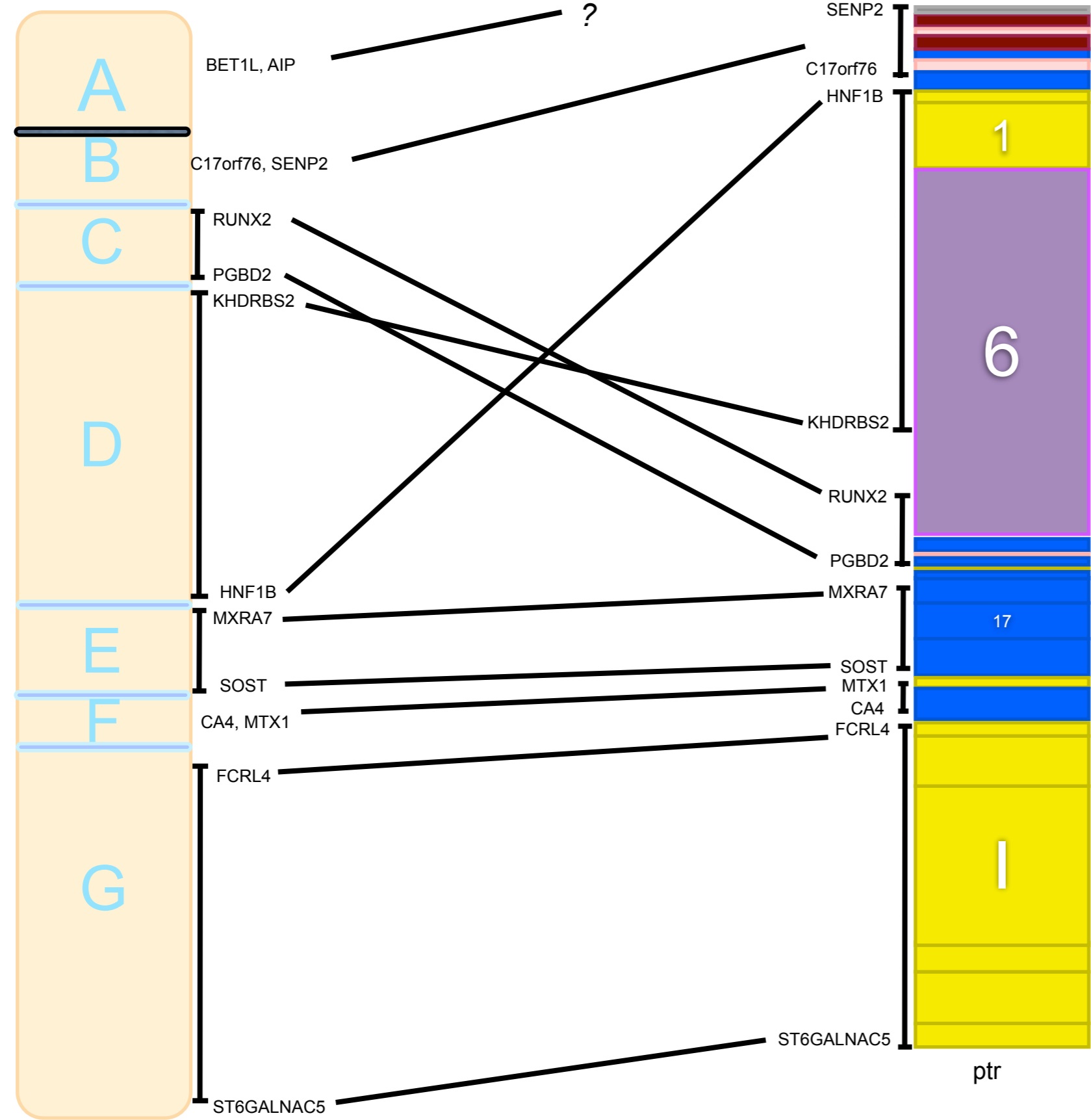

Supplement: Additional file 2 — Figure S2. Comparative map between tammar wallaby chromosome 2 (MEU2) and opossum chromosome 2 (MDO2). Note: For easier comparison with MEU2, MDO2 is shown with its q telomere at the top and its p telomere at the bottom. [file 1471-2164-12-422-S2.PDF]

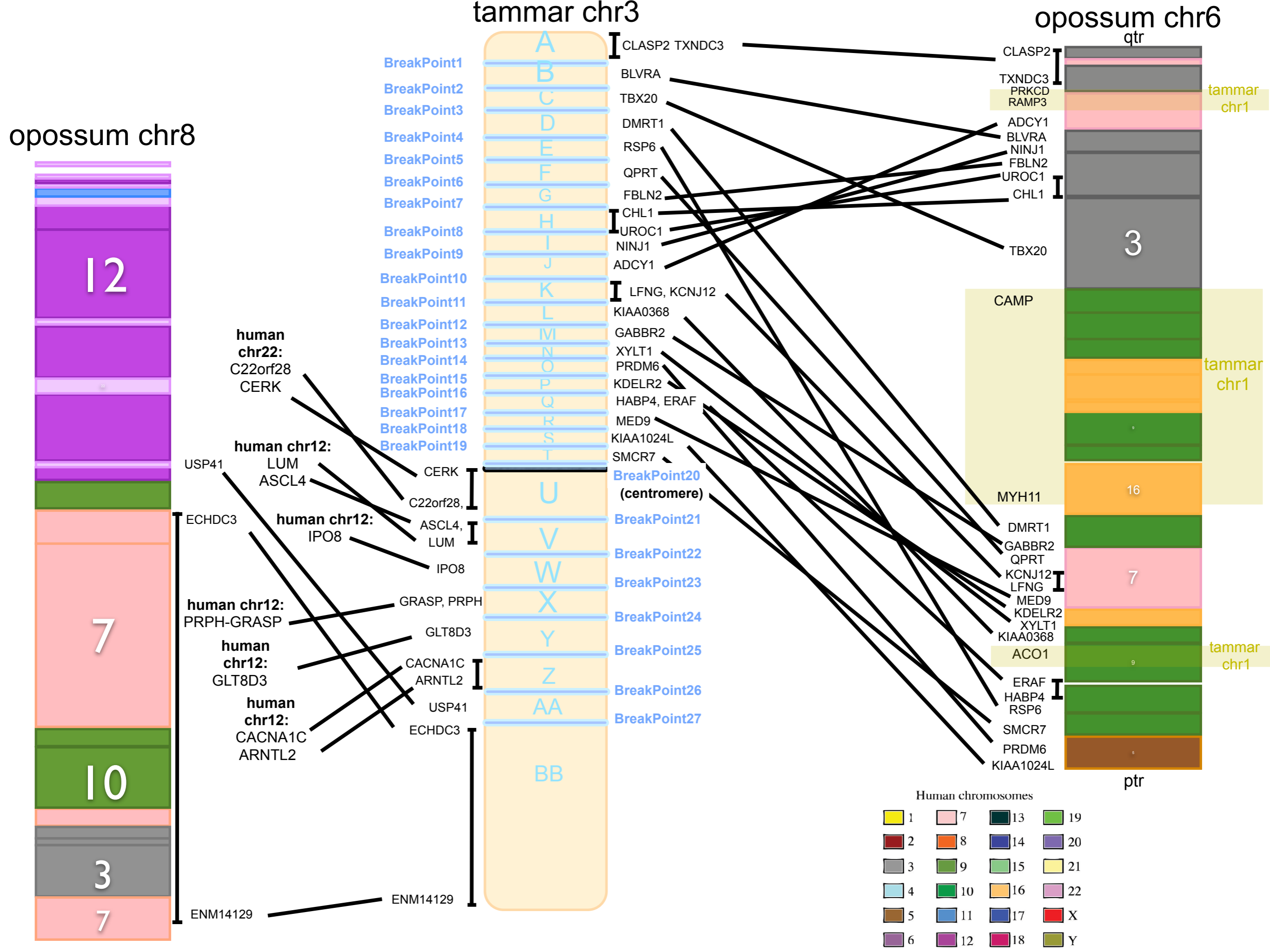

Supplement: Additional file 3 — Figure S3. Comparative map between tammar wallaby chromosome 3 (MEU3) and opossum/human chromosomes (MDO6, MDO8, HSA12, HSA22). Note: For easier comparison with MEU3, MDO6 is shown with its q telomere at the top and its p telomere at the bottom. [file 1471-2164-12-422-S3.PDF]

# tammar chr4

# opossum chr3

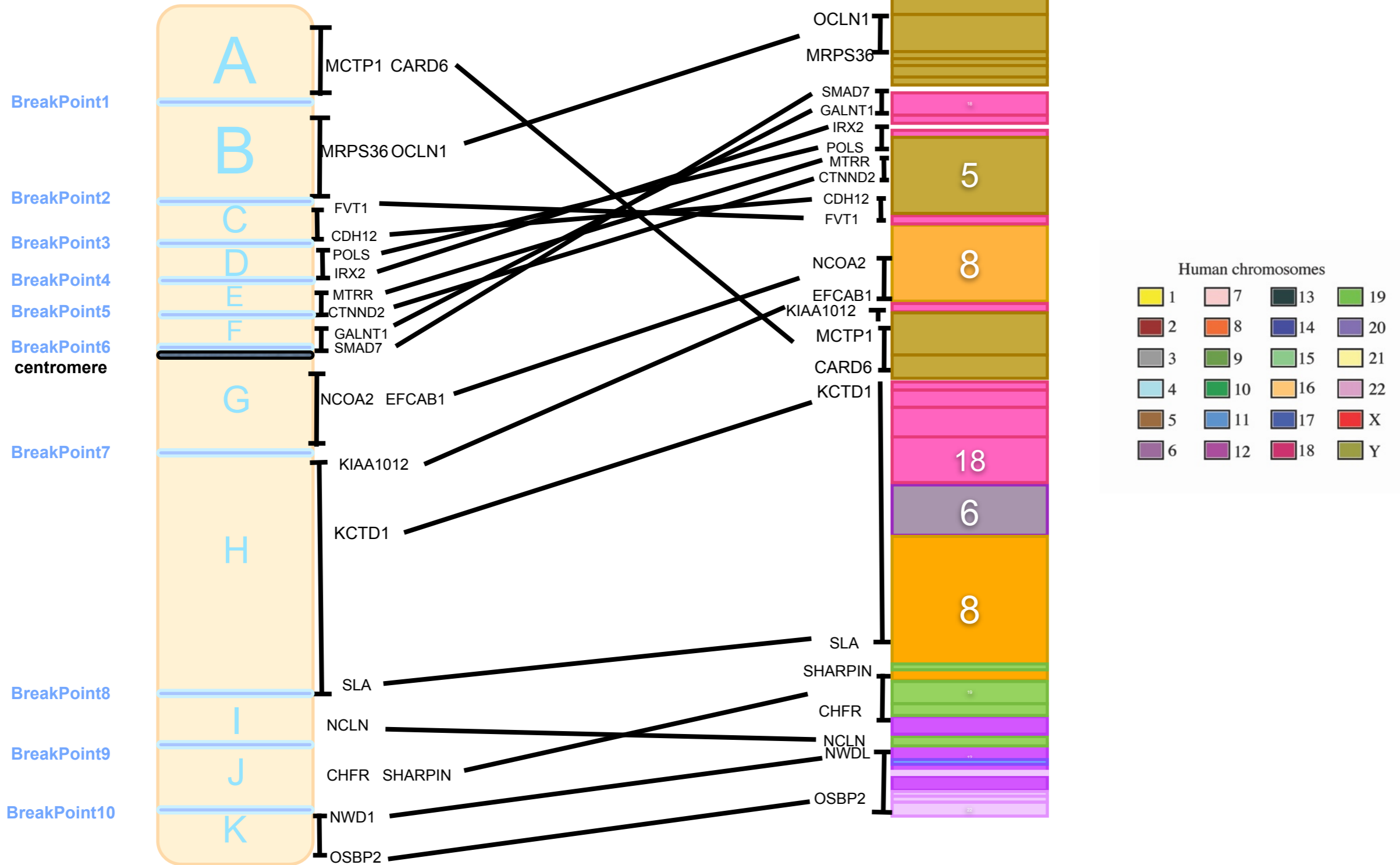

Supplement: Additional file 4 — Figure S4. Comparative map between tammar wallaby chromosome 4 (MEU4) and opossum chromosome 3 (MDO3). [file 1471-2164-12-422-S4.PDF]

tammar chr6

## opossum chr5

## opossum chr7

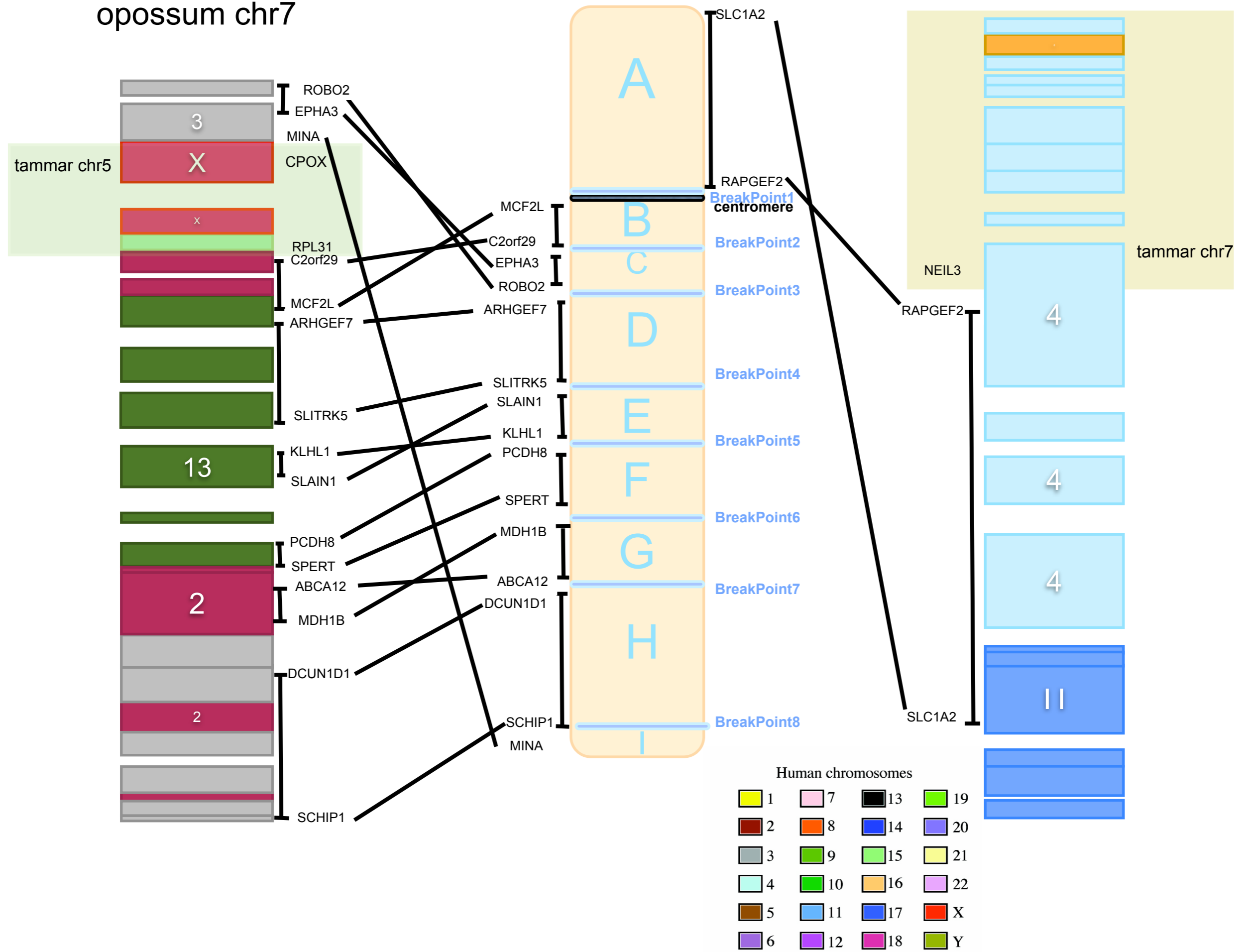

Supplement: Additional file 5 — Figure S5. Comparative map between tammar wallaby chromosome 6 (MEU6) and opossum chromosomes 5 and 7 (MDO5, MDO7). [file 1471-2164-12-422-S5.PDF]
