# Supplementary material for: A first-generation integrated tammar wallaby map and its use in creating a tammar wallaby first-generation virtual genome map
Source: BMC Genomics. 2011 Aug 19;12:422. doi: 10.1186/1471-2164-12-422 (PMC3170641; doi:10.1186/1471-2164-12-422)
Supplement: Additional file 6 — Figure S6. Comparative map between tammar wallaby chromosome 7 (MEU7) and opossum chromosomes 1 and 5 (MDO1, MDO5). [file 1471-2164-12-422-S6.PDF]

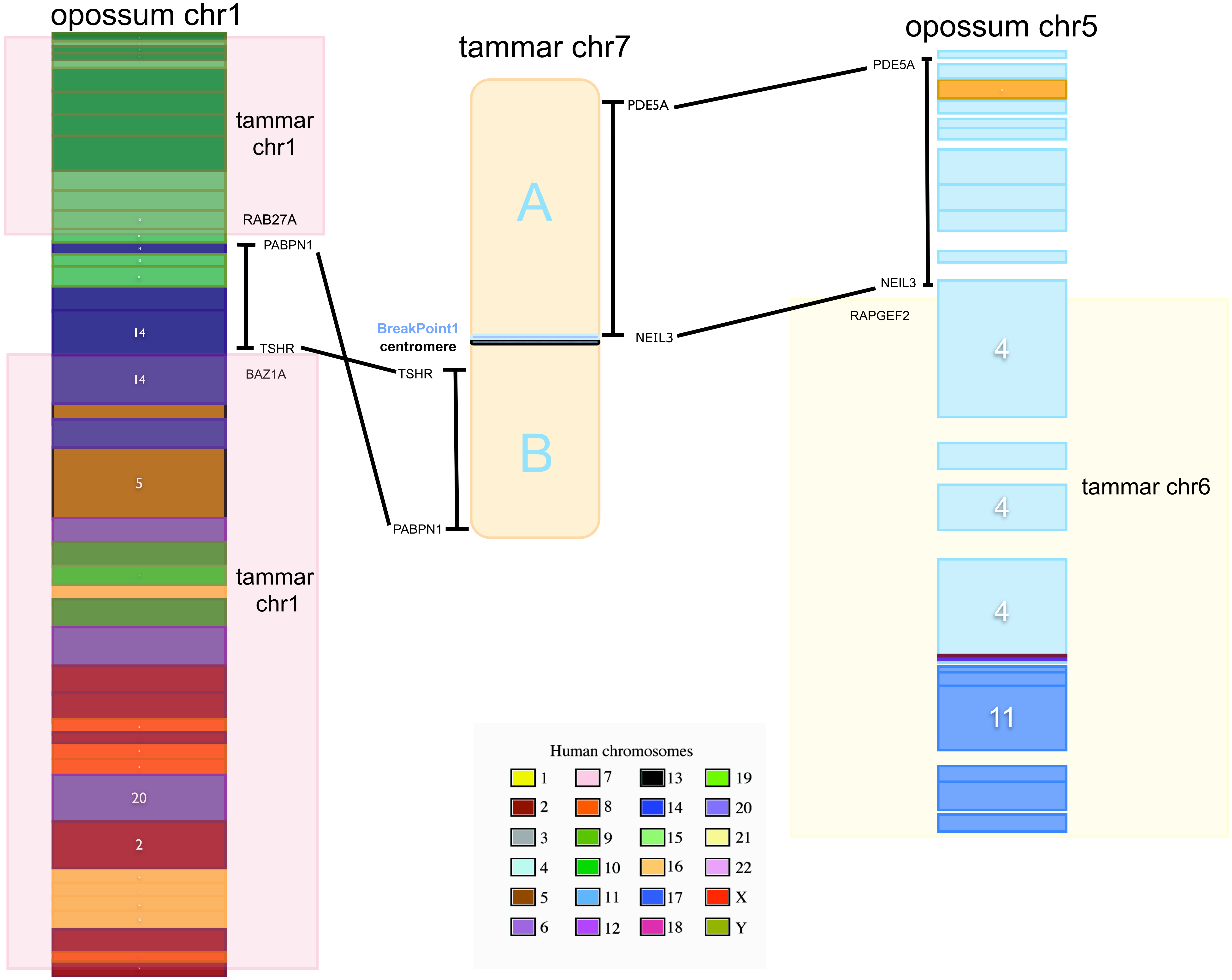
opossum chr1
tammar chr1
RAB27A
PABPN1
14
TSHR
14
BAZ1A
5
tammar chr1
20
2
tammar chr7
A
BreakPoint1
centromere
TSHR
B
PABPN1
PDE5A
NEIL3
opossum chr5
PDE5A
NEIL3
RAPGEF2
4
tammar chr6
4
4
11
Human chromosomes
1
2
3
4
5
6
7
8
9
10
11
12
13
14
15
16
17
18
19
20
21
22
X
Y
