# Supplementary material for: A first-generation integrated tammar wallaby map and its use in creating a tammar wallaby first-generation virtual genome map
Source: BMC Genomics. 2011 Aug 19;12:422. doi: 10.1186/1471-2164-12-422 (PMC3170641; doi:10.1186/1471-2164-12-422)
Supplement: Additional file 1 — Figure S1. Comparative map between tammar chromosome 1 (MEU1) and opossum chromosomes 1 and 6 (MDO1, MDO6). Note: For easier comparison with MEU1, MDO6 is shown with its q telomere at the top and its p telomere at the bottom. [file 1471-2164-12-422-S1.PDF]

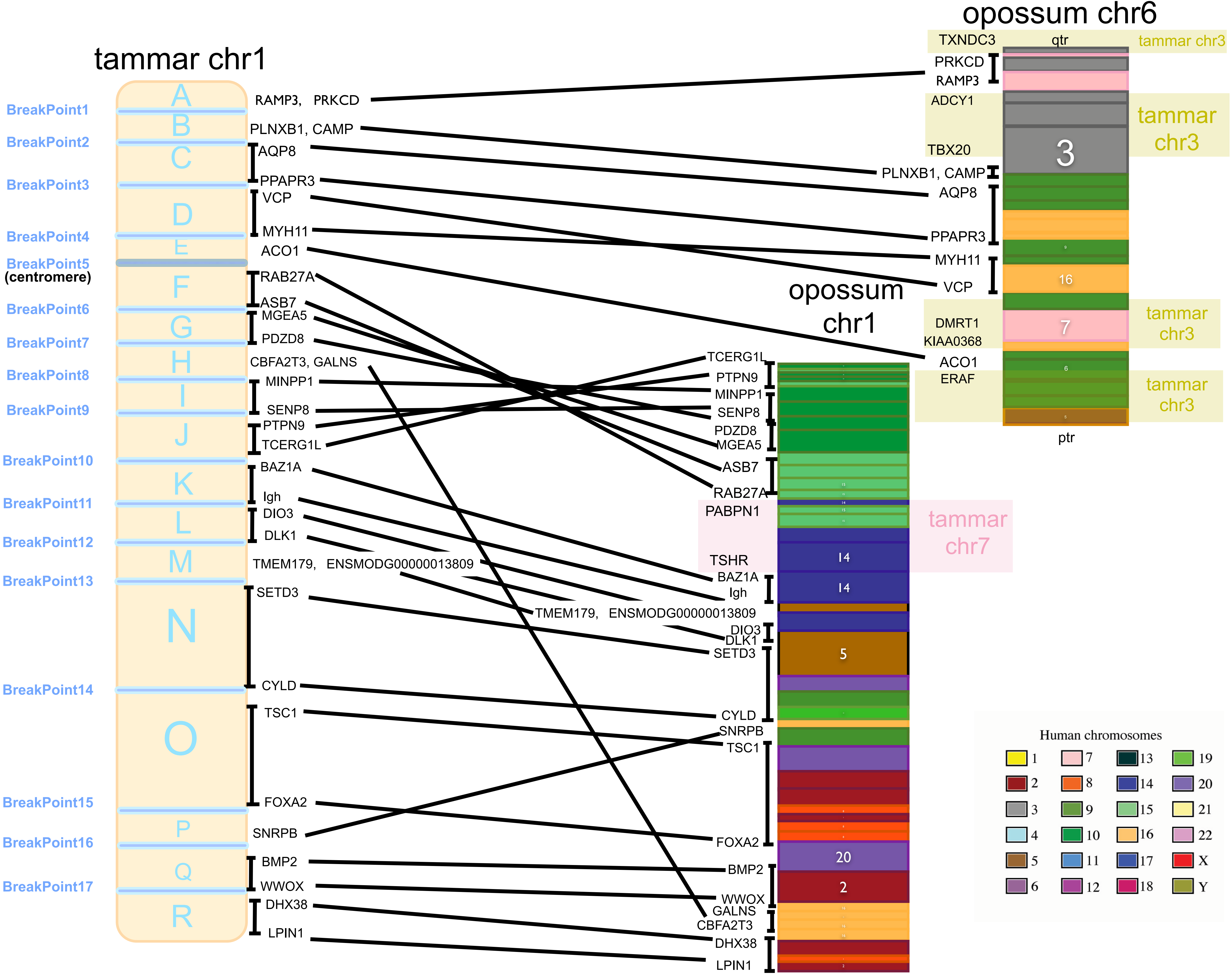

opossum chr6
tammar chr1
opossum chr1
BreakPoint1
BreakPoint2
BreakPoint3
BreakPoint4
BreakPoint5
(centromere)
BreakPoint6
BreakPoint7
BreakPoint8
BreakPoint9
BreakPoint10
BreakPoint11
BreakPoint12
BreakPoint13
BreakPoint14
BreakPoint15
BreakPoint16
BreakPoint17
A
B
C
D
E
F
G
H
I
J
K
L
M
N
O
P
Q
R
RAMP3, PRKCD
PLNXB1, CAMP
AQP8
PPAPR3
VCP
MYH11
ACO1
RAB27A
ASB7
MGEA5
PDZD8
CBFA2T3, GALNS
MINPP1
SENP8
PTPN9
TCERG1L
BAZ1A
Igh
DIO3
DLK1
TMEM179, ENSMODG00000013809
SETD3
CYLD
TSC1
FOXA2
SNRPB
BMP2
WWOX
DHX38
LPIN1
TXNDC3
qtr
tammar chr3
PRKCD
RAMP3
ADCY1
TBX20
tammar chr3
3
PLNXB1, CAMP
AQP8
PPAPR3
MYH11
VCP
16
DMRT1
KIAA0368
7
tammar chr3
ACO1
ERAF
tammar chr3
ptr
TCERG1L
PTPN9
MINPP1
SENP8
PDZD8
MGEA5
ASB7
RAB27A
PABPN1
tammar chr7
TSHR
14
BAZ1A
14
Igh
TMEM179, ENSMODG00000013809
DIO3
DLK1
SETD3
5
CYLD
SNRPB
TSC1
FOXA2
20
BMP2
2
WWOX
GALNS
CBFA2T3
DHX38
LPIN1
Human chromosomes
1
2
3
4
5
6
7
8
9
10
11
12
13
14
15
16
17
18
19
20
21
22
X
Y
